# *Biomedicines*—Moving Biologic Agents into Approved Treatment Options

**DOI:** 10.3390/biomedicines1010001

**Published:** 2013-03-11

**Authors:** Kenneth Cornetta

**Affiliations:** Department of Medical and Molecular Genetics, Indiana University School of Medicine, 975 W, Walnut Street Room 130, Indianapolis, IN 46202, USA; E-Mail: kcornett@iu.edu

The development of biologic agents for therapeutic purposes, or biomedicines, has seen an active area of research both at the bench and in clinical trials. There is mounting evidence that biologic products can provide effective therapy for diseases that have been unresponsive to traditional pharmacologic approaches. Monoclonal antibody therapy for cancer and rheumatologic diseases has become a well accepted part of disease treatment plans. Gene therapy products have been approved in China and Europe. Bioengineering of new agents capitalizing on microRNA biology, nanoparticle technology, stem cell biology, and an increasing understanding of immunology predict a rich future for product development.

The journal *Biomedicines* seeks to provide a home for publication of high quality manuscripts that span the field of biologic agents. These include vaccines, monoclonal antibodies, immunotherapy, cell therapeutics, and gene therapy. While it may appear that these agents are distinct, they are in fact linked in many ways. Vaccine development using attenuated virus strains has provided important lessons for the development of viral gene therapy. In turn, the tools developed in gene therapy have fostered genetic manipulations that have improved traditional vaccines. Gene therapy relies heavily on the advances developed in the cell therapy field. In turn, gene transfer tools were critical in the first successful development of induced pluripotent stem cells. Proof of principle for the successful immunotherapy of cancer has relied on our understanding of antibody recognition, gene transfer, and cell-based therapy. A journal focused on the novelty, complimentary, and often inter-dependent nature of biologic products is the goal of *Biomedicines*.

A novel aspect of *Biomedicines* is an understanding that developing biologic agents requires an extensive community of scientists and clinicians. Bioengineers, basic biologists, virologists, immunologists, and clinicians all contribute to identifying disease targets and developing the technologic capabilities to deliver novel therapeutics. *Biomedicines* seeks to publish important studies that include basic scientific studies, product development, product manufacture and clinical trials. Furthermore, the regulatory environment for many novel agents is uncharted and both cellular and genetic therapies raise ethical issues that must be addressed if these products are to move into the clinic. All these issues are within the scope of *Biomedicines*.

As I look forward, I am excited to serve as the Editor-in-Chief of *Biomedicines* and collaborate with an excellent editorial board with outstanding experience across the spectrum of novel biologic therapies. Our plan is to start the journal off with a series of focused issues that highlight the scope of the new journal. I am particularly excited about the role *Biomedicines* can play in advancing the goals of our multi-disciplinary community in developing and implementing novel biologic agents. I am particularly drawn to the online access aspect of the new journal that will make manuscripts published in *Biomedicines* available worldwide at no cost to the reader.

On behalf of the editorial board I encourage you to participate in this exciting new journal.

